# Microarray Analysis of the Juvenile Hormone Response in Larval Integument of the Silkworm, *Bombyx mori*

**DOI:** 10.1155/2014/426025

**Published:** 2014-04-06

**Authors:** Daojun Cheng, Jian Peng, Meng Meng, Ling Wei, Lixia Kang, Wenliang Qian, Qingyou Xia

**Affiliations:** ^1^State Key Laboratory of Silkworm Genome Biology, Southwest University, No. 2, Tiansheng Road, Beibei District, Chongqing 400715, China; ^2^School of Life Science, Southwest University, Chongqing 400715, China

## Abstract

Juvenile hormone (JH) coordinates with 20-hydroxyecdysone (20E) to regulate larval growth and molting in insects. However, little is known about how this cooperative control is achieved during larval stages. Here, we induced silkworm superlarvae by applying the JH analogue (JHA) methoprene and used a microarray approach to survey the mRNA expression changes in response to JHA in the silkworm integument. We found that JHA application significantly increased the expression levels of most genes involved in basic metabolic processes and protein processing and decreased the expression of genes associated with oxidative phosphorylation in the integument. Several key genes involved in the pathways of insulin/insulin-like growth factor signaling (IIS) and 20E signaling were also upregulated after JHA application. Taken together, we suggest that JH may mediate the nutrient-dependent IIS pathway by regulating various metabolic pathways and further modulate 20E signaling.

## 1. Introduction

Juvenile hormone (JH) is a sesquiterpenoid hormone that cooperates with 20-hydroxyecdysone (20E) to regulate many aspects of insect physiology, including growth, development, and reproduction [1, 2]. In insects, JH is generally synthesized by the corpora allata and contributes to maintain larval growth [2, 3]. 20E is transformed from ecdysone produced in the prothoracic glands and triggers larval molting and metamorphosis with the larval-pupal transition. Higher levels of JH during larval stages prevent metamorphosis, whereas lower levels or an absence of JH at the end of the larval or pupal stages allows 20E to promote metamorphosis [4–6]. JH also regulates female reproductive maturation in adult insects [6].

The mechanism mediating the JH response is a fascinating question. Recently, some studies have reported that JH can induce the transcription of a large number of genes in vivo or in vitro [7–14], and specific JH-response elements (JHREs) in some JH-regulated genes have been identified [10]. However, little is known about the genomewide response to JH and the crosstalk between JH and 20E in insect larvae. Importantly, a transcription factor* Krüppel homolog 1* (*Kr-h1*) is transcriptionally regulated by JH and is involved in transducing the JH signal as a repressor of insect metamorphosis [15–19]. The binding motif of Kr-h1 in the promoter of Kr-h1 target has been identified [20]. Therefore,* Kr-h1* is regarded as an excellent indicator of JH sensitivity in insects [21].

Generally, insects feed and grow during their larval stages. JH is involved in maintaining metabolic homeostasis in insects, which is consistent with its function of keeping insects in the larval growth phase. For example, early evidence from the silkworm (*Bombyx mori*) showed that JH analogue (JHA) application impairs ATP production [22]. Recent reports found that JH regulates lipolysis and trehalose homeostasis in insects [13, 23, 24]. Interestingly, an evolutionarily conserved insulin/insulin-like growth factor signaling (IIS) pathway is also involved in mediating nutrition signaling during insect growth [25]. The IIS pathway participates in the nutritional regulation of JH biosynthesis in the yellow fever mosquito (*Aedes aegypti*) [26–28]. In addition, JH controls trehalose homeostasis to modulate starvation resistance by regulating the synthesis of insulin-like peptide 2 (ILP2) in the red flour beetle (*Tribolium castaneum*) [24]. However, our understanding of the metabolic pathways regulated by JH and their interconnections with the IIS pathway is limited.

The JH titer is high in the early stage of each larval instar whereas the 20E titer is elevated at the later stage. This fluctuation suggests an essential interplay between JH and 20E. To our current knowledge, some molecules, including Met, EcR, Broad complex, and Kr-h1, have been confirmed to orchestrate the crosstalk between JH and 20E during insect growth and development [9, 16, 29–31]. Intriguingly, several studies found that the IIS pathway regulates ecdysteroidogenesis [32–34]. These findings raise the possibility that JH may affect the 20E levels by modulating the activity of the nutrition-dependent IIS pathway.

The silkworm is an excellent model for studying crosstalk between JH and 20E because of the obvious boundary between feeding and molting during larval stages. There are several reports that focus on JH actions in the silkworm. For example, JH has been demonstrated to be a potent stimulator of the secretion of PTTH, a peptide that promotes ecdysteroidogenesis [35]. The overexpression of the juvenile hormone esterase gene that is involved in JH metabolism, the mutation of the* Cyp15C1* gene that is involved in JH biosynthesis, or the direct downregulation of JH biosynthesis in the silkworm result in precocious metamorphosis and a reduction in larval molting times [36–38]. The application of the JHA methoprene at the beginning of the fourth larval instar induces perfectly the emergence of superlarvae with an additional larval instar [39]. JH is also involved in glycolysis and the immune response [11, 13, 40]. However, the genomewide response to JH and the genetic basis for the crosstalk between JH and other signaling pathways, such as the 20E signaling and IIS pathways, are still unclear in the silkworm.

To uncover the genomewide response to JH in vivo and to find new clues to the effect of JH on the signaling pathways related to larval growth and development, we investigated the genomewide changes of gene expression following JHA treatment. We measured gene expression in the integument (including muscle) of the silkworm using a silkworm microarray [41] and a silkworm superlarvae model induced by JHA application [39]. Our results show that JHA application increases the expression levels of genes involved in various metabolic processes and the IIS pathway in the silkworm.

## 2. Materials and Methods

### 2.1. Silkworm Strain

The silkworm strain* Dazao* (p50) with a characteristic of tetramolter (four molting times during larval stages) was used in our experiments. The larvae were reared on fresh mulberry leaves at 25°C under a 12-hour light/12-hour dark photoperiod.

### 2.2. JHA Induction of Silkworm Superlarvae

We collected individual larvae one hour after the completion of the third larval molt and reared together to use for hormone treatment experiments. The JHA methoprene (Sigma, USA) was dissolved in acetone. According to previous report [35, 38, 39], doses of 10–30 *μ*g per larva were applied to newly molted fourth instar larvae along the dorsal midline of the larval thorax. Silkworm larvae treated with the same dose of pure acetone were used as a control.

### 2.3. Microarray Analysis

Larval integument (including muscle) is considered a peripheral tissue targeted by JH in insects [42]. To analyze the genomewide responses to JHA application in the silkworm integument, we separately isolated the integument from 20 larval individuals at different time points after JHA treatment, including 12 hours after treatment (Hat) with JHA or acetone (control), 24 Hat, 36 Hat, and 48 Hat. Integument samples at each time point were immediately snap-frozen in liquid nitrogen and stored at −80°C. Three independent samples for each time point were isolated as biological replicates.

We further surveyed the changes of gene expression after JHA application using silkworm genomewide microarray, which has been widely used to profile gene expression in many studies [41, 43]. Total RNA extraction, microarray hybridization, raw data normalization, and microarray data analysis were performed as described in previous reports [40, 41]. In brief, total RNA was extracted from the prepared sample using TRIzol reagent (Invitrogen, USA). Microarray hybridization and raw data normalization were performed by CapitalBio Corp. (China). Because of the high cost and the high reproducibility of microarray hybridization [41], we mixed equal amounts of total RNA from three biological replicates at each time point to create one sample for the hybridization experiment. cDNA for the microarray hybridization was prepared from each mixed RNA template, labeled with fluorescent dyes (Cy5 for the test samples and Cy3 for the control samples), and finally hybridized to the silkworm genome oligonucleotide microarray [41]. The raw signal intensity data for gene expression was extracted from the image obtained via scanning each hybridized array and normalized to the confirmed housekeeping genes.

For the microarray data analysis, we concluded that a gene was expressed in any sample at any time point if its expression signal intensity was greater than 400 units. The ratio of expression change for a gene in each time point after JHA application was evaluated by comparing with that in the control. This ration of expression change for a gene at each time point was further used to profile its time course expression change. Finally, a gene was considered as up- or downregulated after JHA application if it displayed at least 2.0-fold change in expression level compared with the control. All these differentially expressed genes were defined as JHA-modulated genes. Hierarchical clustering of gene expression was performed using the Cluster 3.0 program [44]. To analyze parallel gene expression changes of the JHA-modulated genes across four selected time points, we performed a coexpression analysis using the GeneCluster 2.0 program [45]. All of the microarray data presented in this study have been deposited in the GEO database under accession number GSE53374.

### 2.4. GO Annotation and KEGG Pathway Analysis

Using the online WEGO program [46], we functionally predicted the gene ontology (GO) terms of the JHA-modulated genes. In addition, we used the probe sequences of all up- and downregulated genes to BLAST search against the silkworm gene collection to obtain the probe-matched genes. The online KEGG pathway database (http://www.genome.jp/kegg/) and sequence similarities enabled these probe-matched genes to be mapped to different KEGG pathways using the KAAS (KEGG Automatic Annotation Server) tool [47].

### 2.5. Quantitative Real-Time RT-PCR Experiment

Using quantitative real-time RT-PCR experiments, we examined the changes in the mRNA expression of selected genes in the integument at 12, 24, 36, and 48 hours after JHA application. For an additional comparison, we evaluated the expression of the same gene collection in another JH-targeted tissue, the fat body, after JHA application. Because dissecting the fat body at the early stage of the fourth larval instar is difficult, we only dissected the fat body at 36 and 48 hours after JHA application. Three biological replicates were conducted. Sample collection, RNA extraction, and cDNA synthesis were performed according to previously described procedures [40].

Real-time RT-PCR was performed with the SYBR Premix Ex Taq system (TaKaRa Biotech, Japan). Each PCR reaction was conducted in a final volume of 20 *μ*L containing 70 ng of cDNA (2 *μ*L), SYBR Premix Ex Taq (10 *μ*L), and 0.4 *μ*M primers. The PCR amplification conditions were set to the following: 95°C for 30 s, followed by 40 cycles of 95°C for 30 s and 60°C for 30 s. The mRNA expression levels of the selected genes were normalized to the control of translation initiation factor 4A (*eIF-4A*) gene. All primers used in this study are listed in Table S1. The Pearson correlation coefficient between microarray data and real time RT-PCR results was further calculated.

### 2.6. Prediction of Conserved Binding Sites of Kr-h1 on the Promoters of JHA-Induced Genes

We fetched the sequences from the approximately 3 kb upstream untranslated region (UTR) of translation initiation site of genes that their mRNA expressions were upregulated with at least 1.5-fold at 12 hours after JHA application. These sequences were used to search the conserved recognition and binding motif of transcription factor Kr-h1, a key transducer of JH signal, by the online MatInspector program (http://www.genomatix.de/) [20, 48].

## 3. Results

### 3.1. Induction of Silkworm Superlarvae by JHA Application

**Figure 1:**
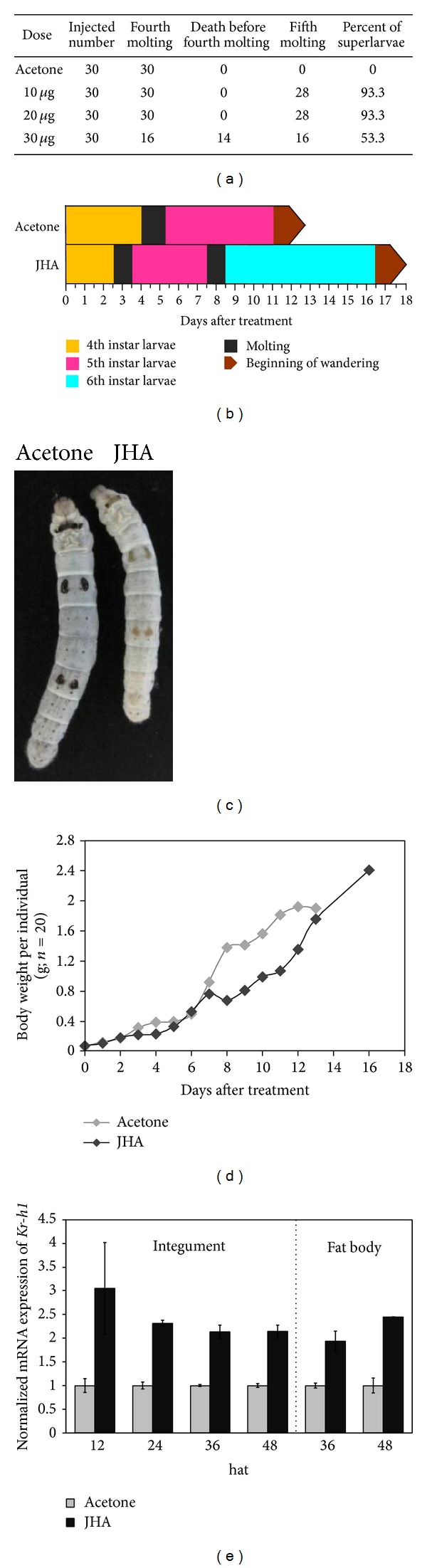
Induction of silkworm superlarvae by JHA application. Silkworm superlarvae were induced by applying the JH analogue (JHA) methoprene to newly molted silkworm larvae at the fourth instar. Silkworm larvae treated with pure acetone were used as a control. (a) Dose-dependent effects of JHA application on silkworm larval molting and survival. (b) JHA application at a dose of 20 *μ*g per larva results in precocious larval molting and induces silkworm superlarvae. (c) Comparison of normal larva with pure acetone treatment (left) and larva with precocious onset of the fourth larval molting at 48 hours after JHA application. The developmental time point is at approximately 60–72 hours after treatment. (d) JHA application changes the body weight of silkworm larvae. (e) The mRNA expression of* Kr-h1*, a JH-responsive gene, is increased after JHA application. Hat: hours after treatment.

To determine the gene expression changes in silkworm in response to JH, we used the JHA methoprene to induce silkworm superlarvae according to previous reports [38, 39]. After several doses of JHA were applied topically to newly molted fourth instar larvae, we found that 10 or 20 *μ*g of JHA per larva induced silkworm superlarvae with a high probability of 93.3%. However, a dose of 30 *μ*g per larva resulted in approximately 50% larval death before the fourth molting (Figure 1(a)).

Therefore, we applied 20 *μ*g of JHA per larva to hundreds of silkworm larvae for further analysis. Compared with pure acetone treatment as control, the JHA application initiated premature fourth molting and led to an additional fifth molting, with the consequence of becoming superlarvae (Figures 1(b) and 1(c)). Although the developmental duration of the fourth and fifth larval instars of silkworm superlarvae was shortened owing to an additional sixth larval instar, the entire larval stage was extended by approximately six days, and the average weight per individual was elevated before wandering (Figure 1(d)). Furthermore, quantitative RT-PCR examination showed that in silkworm superlarvae, the JH-regulated marker gene* Kr-h1* displayed a significantly upregulated expression in the integument at 12, 24, 36, and 48 Hat (Figure 1(e)) and in the fat body at 36 and 48 Hat.

### 3.2. JHA Application Induced a Global Alteration of Gene Expression

**Figure 2:**
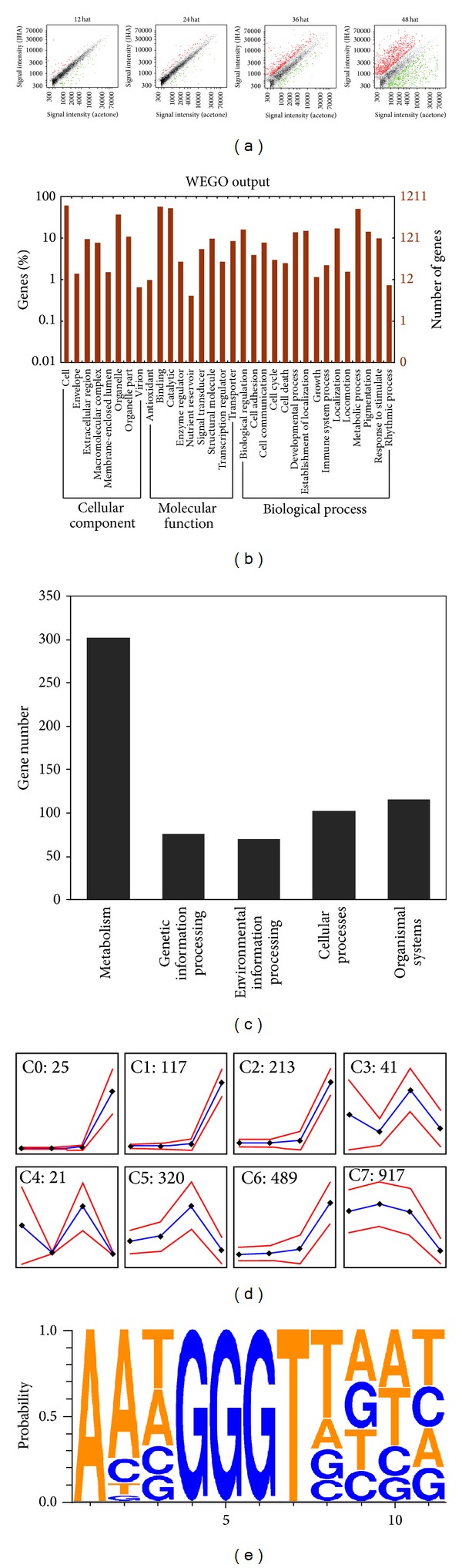
Genomewide alteration of gene expression after JHA application. Genomewide microarray analysis revealed that gene expressions in the integument and fat body were altered after JHA application. (a) Scatter plot of genomewide changes in gene expression. Hat: hours after treatment. (b) Gene ontology (GO) annotation of JHA-modulated genes with an at least 2.0-fold change in expression level after JHA application. All JHA-modulated genes are listed in Table S3 available online at http://dx.doi.org/10.1155/2014/426025. (c) JHA-modulated genes can be functionally classified into different KEGG pathways. For details see Tables S5 and S8. (d) Time course-dependant coexpression of JHA-modulated genes after JHA application in the integument. The digits denote the number of JHA-modulated genes in each cluster. The JHA-modulated genes in each cluster are listed in Table S6. (e) Multiple alignments of conserved binding motifs of Kr-h1 within the upstream UTR regions of JHA-induced genes.

Based on the model of JHA-induced silkworm superlarvae, we used a genomewide silkworm microarray to analyze the global gene expression pattern in the integument following JHA application. As shown in Table S2 and Figure S1, a total of 8,670 genes were expressed in the integuments with both treatments from JHA and pure acetone. Scatter plot analysis demonstrated that the expression levels of many genes were altered by JHA application (Figure 2(a)). Intriguingly, 2,143 genes exhibited an expression change of at least 2.0-fold after JHA application (Table S3). For convenience in the following description, we defined these genes as JHA-modulated genes. In detail, at 12 Hat, 49 and 87 genes were up- and downregulated, respectively. At 24 Hat, 69 and 74 genes were up- and downregulated, respectively. At 36 and 48 Hat, respectively, 450 and 857 genes were upregulated, while 165 and 956 genes were downregulated. Further real-time RT-PCR experiments confirmed that most of the examined genes, whose expressions were deregulated after JHA application, showed good correlations (Pearson correlation coefficient, *r* > 0.7) between microarray data and RT-PCR results (Table S4).

GO annotation showed that JHA-modulated genes could be functionally classified into 32 categories (Figure 2(b)). The number of JH-modulated genes belonging to the categories of catalytic activity and metabolic process was the largest. In addition, KEGG analysis revealed that JHA-modulated genes were mainly related to metabolism, genetic information processing, environmental information processing, cellular processes, and organismal systems (Figure 2(c); Table S5). Remarkably, after a redundancy subtraction for probes, we noted that JHA-modulated genes involved in basic metabolic processes have the largest number (i.e., 302).

GeneCluster-based coexpression analysis revealed that the expression changes of most JHA-modulated genes in the integument exhibited a similar time course dependence. As shown in Figure 2(d) and Table S6, the dynamic expression of JHA-modulated genes could be grouped into eight clusters. JHA-modulated genes from the C0, C1, C2, and C6 clusters were upregulated at 48 Hat. The C3 and C4 clusters showed an upregulation at 12 and 36 Hat as well as a downregulation at 24 and 48 Hat. C5 was upregulated at 36 Hat and then downregulated at 48 Hat. C7 showed a significant downregulation from 12 to 48 Hat.

We used MatInspector program to search the binding motifs of transcription factors in the upstream UTR regions of the translation start sites of 157 genes whose expression showed upregulation with at least 1.5-fold at 12 Hat after JHA application. Our results found that the conserved binding motif of Kr-h1, which is a key transcription factor for mediating JH signal, was present within 3 kb upstream UTR regions of 95 of the 157 JHA-induced genes at 12 Hat (Table S7). Importantly, as shown in Figure 2(e), the Kr-h1 binding motifs of 67 JHA-induced genes revealed the highest core similarity (Core sim) of 1 with the binding motif of Kr-h1 [20], which all were comprised of a consensus sequence GGGT. This indicates that these JHA-induced genes may be the direct targets of silkworm Kr-h1.

### 3.3. JHA Application Facilitates Basic Metabolic Processes

**Figure 3:**
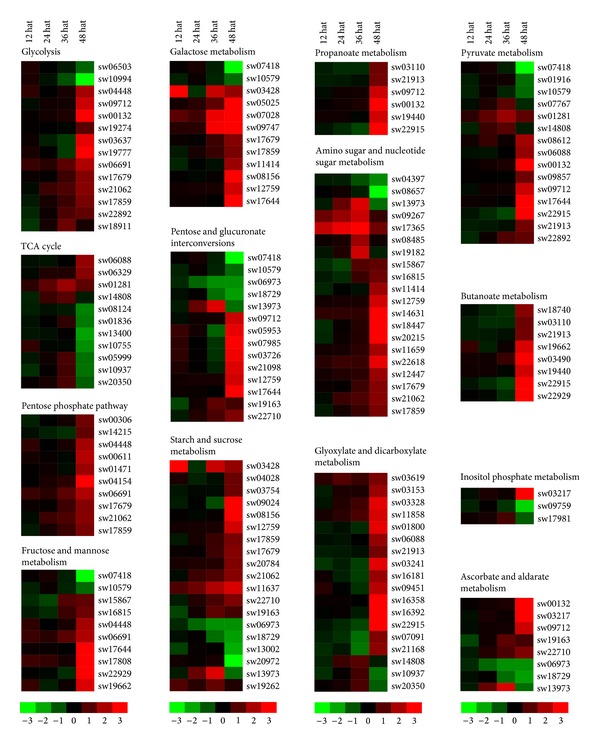
JHA-modulated pathways related to carbohydrate metabolism. Hierarchical clustering of JHA-modulated genes involved in 14 pathways related to carbohydrate metabolism. The probe ID, KEGG ID, and expression intensity of each JHA-modulated gene are listed in Tables S5 and S8. Hat: hours after treatment.

Because the number of JHA-modulated genes involved in metabolic pathways was the highest and the main function of JH is to maintain insect larval growth, we further investigated JHA-modulated genes related to metabolic pathways. Our results from the KEGG analysis showed that 99 JHA-modulated genes were involved in carbohydrate metabolism (Figure 3; Table S8), with 74 genes that were upregulated in the integument after JHA application and that could be classified as members of the C0, C1, C2, C5, or C6 clusters (Table S6). Interestingly, we observed that JHA-modulated genes involved in three pathways of carbohydrate metabolism were all upregulated, including ten genes for the pentose phosphate pathway (e.g., ribose-phosphate pyrophosphokinase, 6-phosphogluconate dehydrogenase, 6-phosphogluconolactonase, phosphoglucomutase, transketolase, fructose-1,6-bisphosphatase I, fructose-bisphosphate aldolase, and glucose-6-phosphate isomerase), five genes involved in propanoate metabolism (i.e., 4-aminobutyrate aminotransferase, aldehyde dehydrogenase, aldehyde dehydrogenase family 7 member A1, acetyl-CoA C-acetyltransferase, and enoyl-CoA hydratase), and five genes involved in butanoate metabolism (i.e., 3-oxoacid CoA-transferase, 4-aminobutyrate aminotransferase, enoyl-CoA hydratase, hydroxymethylglutaryl-CoA lyase, and acetyl-CoA C-acetyltransferase). Similarly, most of the genes implicated in other several carbohydrate metabolism pathways (such as glycolysis, pentose and glucuronate interconversions, galactose metabolism, starch and sucrose metabolism, amino sugar and nucleotide sugar metabolism, pyruvate metabolism, and glyoxylate and dicarboxylate metabolism) were also upregulated by JH application.

**Figure 4:**
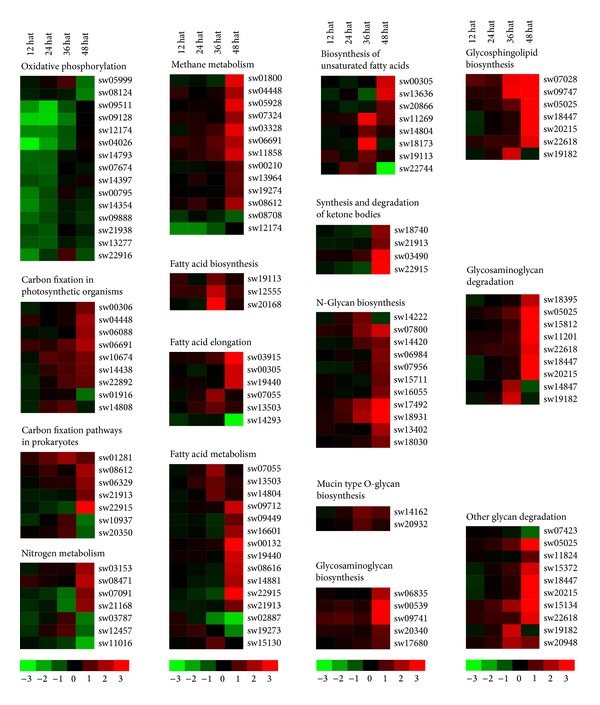
JHA-modulated pathways related to the metabolism of energy, lipids, or glycans. Cluster analysis of JHA-modulated genes involved in five pathways related to energy metabolism, five pathways related to lipid metabolism, and six pathways related to glycan metabolism. The probe ID, KEGG ID, and expression intensity of each JHA-modulated gene are listed in Tables S5 and S8. Hat: hours after treatment.

The KEGG analysis showed that 47 JHA-modulated genes are involved in energy metabolism (Figure 4; Table S8). In addition, 25 JHA-modulated genes were upregulated, with most (18) of them belonging to cluster C6, which is upregulated at 48 Hat. In detail, more than half of the genes involved in each of the four pathways related to energy metabolism, including methane metabolism, nitrogen metabolism, carbon fixation in photosynthetic organisms, and carbon fixation pathways in prokaryotes, were upregulated. Intriguingly, we found that all 15 JH-modulated genes involved in the program of oxidative phosphorylation (OXPHOS) were downregulated, including 13 types of V-type H^+^-transporting ATPase subunits and two succinate dehydrogenase (ubiquinone) subunits.

There are 58 enzyme-encoding genes involved in 14 pathways related to lipid metabolism that were modulated by JHA (Figure 4; Table S8). Of these genes, 38 were upregulated. All 11 genes catalyzing the biosynthesis of fatty acids and unsaturated fatty acids were upregulated after JHA induction, such as fatty acid synthase, 3-oxoacyl-[acyl-carrier protein] reductase, very-long-chain 3-oxoacyl-CoA reductase, stearoyl-CoA desaturase (delta-9 desaturase), and acyl-CoA oxidase. Similarly, among 18 genes involved in the elongation and metabolism of fatty acids, 14 genes were upregulated after JHA application, including acetyl-CoA acyltransferase, palmitoyl-protein thioesterase, aldehyde dehydrogenase, long-chain acyl-CoA synthetase, very-long-chain acyl-CoA dehydrogenase, and enoyl-CoA hydratase. Furthermore, four genes participating in the synthesis and degradation of ketone bodies were also induced by JHA.

Among the 37 JHA-modulated genes involved in glycogen biosynthesis and metabolism, 34 were upregulated after JHA application (Figure 4; Table S8). Notably, all JHA-modulated genes involved in the biosynthesis of several types of glycans (e.g., N-glycan, mucin-type O-glycan, glycosaminoglycan, and lipopolysaccharide) were upregulated. In addition, JH-modulated genes for glycosaminoglycan degradation all increased their expression after JHA application, including alpha-1,2-mannosyltransferase, arylsulfatase B, hexosaminidase, xylosylprotein 4-beta-galactosyltransferase, mannosyl-oligosaccharide alpha-1,2-mannosidase, beta-galactosidase, and oligosaccharyltransferase complex subunit beta.

**Figure 5:**
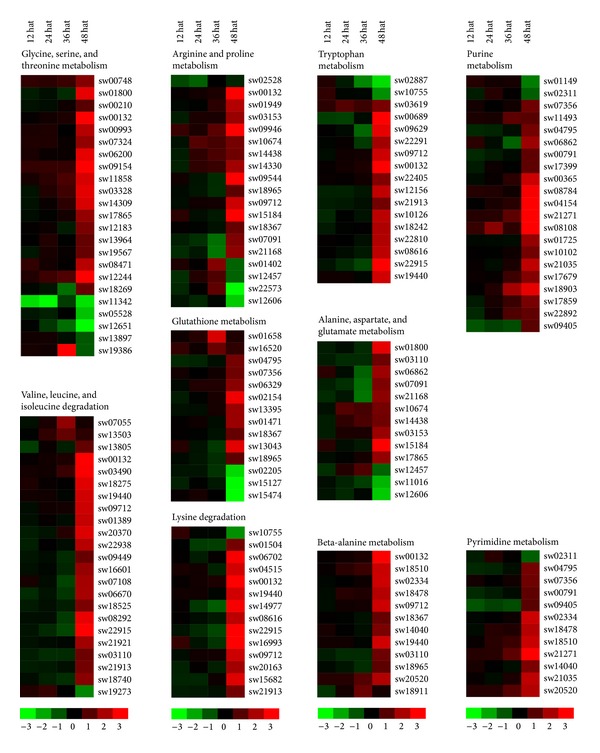
JHA-modulated pathways involved in amino acid and nucleotide metabolisms. Hierarchical clustering of JHA-modulated genes involved in eight pathways related to amino acid metabolism and two pathways related to nucleotide metabolism. The probe ID, KEGG ID, and expression intensity of each JHA-modulated gene are listed in Tables S5 and S8. Hat: hours after treatment.

Our KEGG analysis indicated that 117 JHA-modulated genes were involved in 20 pathways related to amino acid metabolism, with 96 being upregulated after JHA application (Figure 5; Table S8). These pathways involved a large number of JHA-modulated enzymes mainly included the metabolism of glycine, serine, and threonine, the degradation of valine, leucine, and isoleucine, the metabolism of arginine and proline, and the metabolism of tryptophan. The number of JHA-modulated genes for these four pathways was 23, 23, 19, and 17, respectively. In addition, 12 enzyme-coding genes involved in beta-alanine metabolism were all upregulated after JHA application, including dihydropyrimidine dehydrogenase, 4-aminobutyrate aminotransferase, aldehyde dehydrogenase, spermine synthase, beta-ureidopropionase, enoyl-CoA hydratase, and aldehyde dehydrogenase family 7 member A1.

We also noted that 26 JHA-modulated genes were responsible for catalyzing the metabolism of two types of nucleotides: purine and pyrimidine (Figure 5; Table S8). Importantly, most (24) of these genes were upregulated after JHA application, including 5^'^-nucleotidase, adenylate kinase, polyribonucleotide nucleotidyltransferase, urate oxidase, pyruvate kinase, ribose-phosphate pyrophosphokinase, phosphoglucomutase, dihydropyrimidine dehydrogenase, adenylosuccinate lyase, phosphoribosylformylglycinamidine synthase, phosphoribosylaminoimidazole carboxylase, beta-ureidopropionase, purine-nucleoside phosphorylase, and ribonucleoside-diphosphate reductase subunit.

### 3.4. JHA Application Promoted Protein Synthesis and Degradation

**Figure 6:**
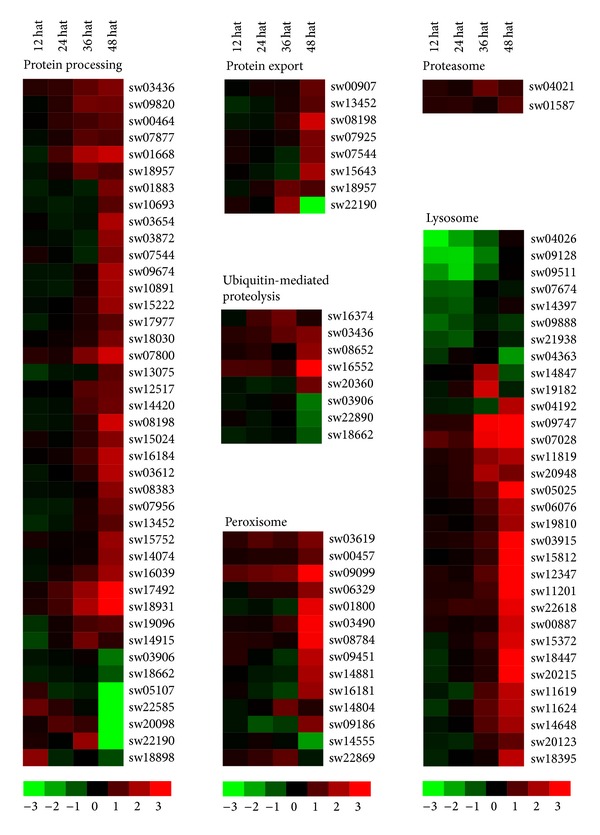
JHA-modulated pathways involved in protein synthesis and degradation. Hierarchical clustering of JHA-modulated genes involved in six pathways related to protein synthesis and degradation, including protein processing, protein export, ubiquitin-mediated proteolysis, proteasome, lysosome, and peroxisome. The probe ID, KEGG ID, and expression intensity of each JHA-modulated gene are listed in Tables S5 and S8. Hat: hours after treatment.

Given that that JH is a key regulator for controlling insect growth and that JHA application enhanced a variety of basic metabolic processes, we evaluated the JHA-induced response of factors involved in protein processing pathways including synthesis and degradation. The KEGG analysis revealed that 97 JHA-modulated genes were involved in protein synthesis and degradation, with 79 being upregulated after JHA application (Figure 6; Table S8). Among the 41 JH-modulated genes involved in protein processing in the endoplasmic reticulum, 34 were upregulated following JHA application, including transitional endoplasmic reticulum ATPase, protein disulfide-isomerase, mannosyl-oligosaccharide alpha-1,2-mannosidase, protein transport protein SEC6, and translocation and translocation-associated proteins.

The expression of eight JHA-modulated genes related to protein export were increased after JHA application (Figure 6; Table S8), including protein transport protein SEC61 subunit alpha, protein transport protein SEC61 subunit gamma, translocation protein SEC63, signal peptidase complex subunit 2, signal recognition particle subunit SRP54, and signal recognition particle receptor subunit alpha.

We noted that the expression levels of most JHA-modulated genes associated with protein degradation were also upregulated after JHA application (Figure 6; Table S8), including five genes related to ubiquitin-mediated proteolysis (i.e., E3 ubiquitin-protein ligase synoviolin, WW domain-containing E3 ubiquitin protein ligase 1, E3 ubiquitin-protein ligase HERC2, DNA damage-binding protein 1, and F-box and WD-40 domain protein), two genes involved in proteasome formation (i.e., 26S proteasome regulatory subunits N1 and N2), 22 genes associated with the lysosome (including four cathepsin family members, hexosaminidase, arylsulfatase B, glucosylceramidase, and beta-galactosidase), and 13 genes associated with the peroxisome (including catalase, phytanoyl-CoA hydroxylase, hydroxymethylglutaryl-CoA lyase, (S)-2-hydroxy-acid oxidase, fatty acyl-CoA reductase, isocitrate dehydrogenase, and superoxide dismutase).

### 3.5. The IIS Pathway Was Increased by JHA Application

**Figure 7:**
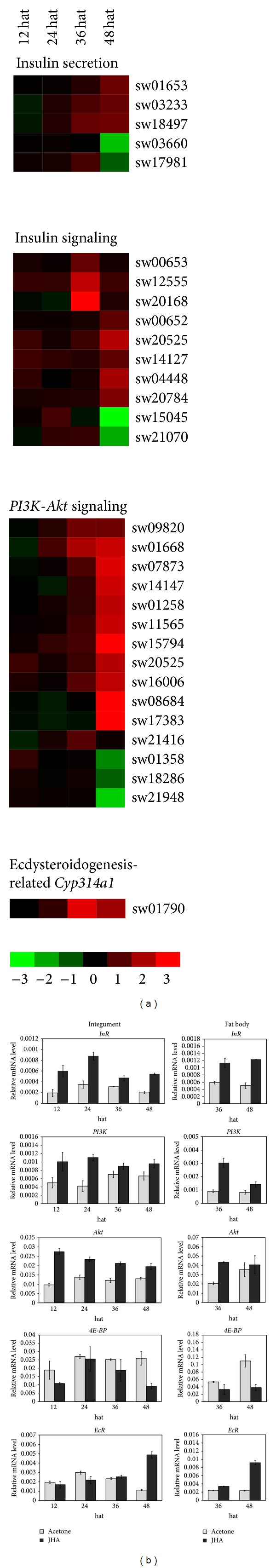
The IIS pathway is modulated by JHA. (a) Hierarchical clustering of JHA-modulated genes related to the IIS pathway and 20E biosynthesis. The probe ID, KEGG ID, and expression intensity of each JHA-modulated gene are listed in Tables S5 and S8. Hat: hours after treatment. (b) Real-time RT-PCR analysis of JHA modulation of the expression levels of key regulators involved in the IIS pathway and 20E signaling.

Intriguingly, our microarray data showed that the expression level of the insulin receptor gene* InR* (probe ID: sw20525) was increased in the integument following JHA application (Figure 7(a); Table S8), which was also confirmed in the integument and fat body using quantitative real-time RT-PCR (Figure 7(b)). We further examined the expression of other JHA-modulated genes related to the IIS pathway in our microarray data. The result showed that 21 of 29 JH-modulated genes involved in the IIS pathway were upregulated after JHA application, including two genes for insulin secretion (i.e., solute carrier family 2 and sodium/potassium-transporting ATPase subunit beta), five genes for insulin signaling (receptor-type tyrosine-protein phosphatase F, starch phosphorylase, fatty acid synthase, fructose-1,6-bisphosphatase I, and protein phosphatase 1-regulatory (inhibitor) subunit 3), and six genes for PI3K-Akt signaling (heat shock protein 90 kDa beta, laminin alpha, laminin beta, laminin gamma, protein phosphatase 2 regulatory subunit A, and thrombospondin). Importantly, real-time RT-PCR examination revealed that three key genes involved in the IIS pathway,* PI3K*,* Akt*, and* 4E-BP*, were also mediated by JHA application (Figure 7(b)). After JHA application,* PI3K* and* Akt* were upregulated, whereas* 4E-BP* was downregulated in both the integument and the fat body.

Given that insulin signaling pathways are implicated in ecdysteroidogenesis in insects [32, 33] and larval molting is controlled by a 20E pulse, it was necessary to check whether genes involved in 20E-related pathways were also upregulated in JHA-induced silkworm superlarvae. As expected, the cytochrome p450 enzyme gene* Cyp314a1 *(*Shade*), which is responsible for converting ecdysone to active 20E, was upregulated by JHA from 24 to 48 Hat (Figure 7(a); Table S8). The RT-PCR examination showed that the 20E receptor gene* EcR* was also upregulated after JHA induction (Figure 7(b)).

## 4. Discussion

The larval stage in most holometabolic insects is characterized by nutrient-dependent growth and several times of larval molting. Insect larval growth and molting are mainly coordinated by two endocrine hormones, JH and 20E. JH maintains larval growth whereas nutrient-sensitive 20E signal triggers the initiation of larval molting. Therefore, larval growth and molting are excellent biological events for studying the crosstalk between JH and 20E in insects. Undoubtedly, insect larval growth must involve various metabolic processes. However, which metabolic processes are regulated by JH during larval growth and how JH contributes to 20E signaling have not been comprehensively examined. In this study, we used the model of JHA-induced silkworm superlarvae and microarray-based gene expression profiling to investigate the functions of JH in both the maintenance of metabolic homeostasis and the 20E-based regulation of larval molting during silkworm larval stages.

Generally, the silkworm larva undergoes four larval molts and forms five larval instars. Previous studies reported that the application of the JHA methoprene to silkworm larvae at the beginning of the fourth larval instar will induce superlarvae with a supernumerary fifth molting [39]. To understand the mechanisms underlying the crosstalk between JH and 20E during the larval stage, we induced silkworm superlarvae by JHA application and observed that the onset of the fourth molt in superlarvae was approximately one day earlier than that in the control larvae treated with pure acetone (Figure 1). The JH-responsive gene* Kr-h1* also showed increased expression in two JH-targeted tissues of superlarvae, the integument and fat body, further confirming that the induction of silkworm superlarvae could be the consequence of JHA application.

To determine how silkworm larvae respond to JH during larval growth and molting, we performed a microarray-based analysis of gene expression changes after JHA application in the integument. A genomewide silkworm microarray has been previously used to profile gene expression in the silkworm [11, 40, 41]. In addition, the identification of JH-responsive genes has been performed in some specific cell lines or tissues as well as at different developmental stages in some insects, including the fruit fly (*Drosophila melanogaster*) [8, 9, 12], yellow fever mosquito [7], red flour beetle [49], pine engraver beetle (*Ips pini*) [14], and silkworm [11, 13]. Comparatively, some new and interesting findings were identified by our microarray analysis of the JH response in the silkworm integument.

Our studies first provide systematic insights into JH modulation on the programs controlling basic metabolism and protein processing during silkworm larval growth and molting. Comparative analysis showed that the expression levels of thousands of genes were significantly changed after JHA application (Table S3). Interestingly, most of these JHA-modulated genes are involved in basic metabolic pathways (e.g., glycolysis, other carbohydrate metabolic programs, and lipid metabolism) and protein processing pathways. Furthermore, most JHA-modulated genes were upregulated after JHA application (Table S8), indicating that JHA application could promote most metabolic processes and protein processing programs. Previous reports have confirmed that a reduction in JH biosynthesis could decrease the levels of carbohydrate and lipid metabolism in the red flour beetle [24] and that JH application could upregulate both the mRNA levels and enzymatic activity of proteins related to glycolysis in the silkworm fat body [13]. Taken together, we propose that a JH-induced increase in basic metabolic activities may produce more nutritional supplies and further facilitate the growth of silkworm larvae.

An interesting observation of our study is that the mRNA level of trehalose-6-phosphate synthase (TPS), an enzyme controlling starch and sucrose metabolism, showed an obvious increase in the silkworm integument after JHA application, which is different from a previous finding showing no significant changes in* TPS* expression in the silkworm fat body after JHA application [24]. This finding raises the prospect that the JH-induced regulation of* TPS* transcription might be tissue specific in the silkworm. Intriguingly, our data also revealed that most of JH-modulated genes that were involved in OXPHOS exhibited significantly reduced expression in the integument (Figure 4; Table S8). Generally, the OXPHOS pathway mediates energy metabolism and produces ATP via the oxidation of nutrients in the mitochondria of animal muscles [50, 51]. Thus, the reduction of expression level of genes involved in the OXPHOS pathway in the silkworm integument before larval molting indicates that OXPHOS-mediated ATP production or requirements might be decreased after JHA application.

The IIS pathway acts as a nutrient sensor in the regulation of metabolic homeostasis and growth [52]. Previous evidences showed that the IIS pathway is stimulated by high glucose and regulates multiple metabolic pathways related to normal growth through various ways, for example, attenuating sugar levels, promoting glycolysis, and synthesizing fatty acids and proteins [53]. Surprisingly, our microarray data revealed that JHA application increased the expression of the insulin receptor gene* InR* in the integument and upregulated other genes involved in IIS-related pathways, including insulin secretion, insulin signaling, and the PI3K-Akt signaling pathway (Figure 7(a)). Real-time RT-PCR examination further confirmed that among several key regulators involved in the IIS pathway,* InR*,* PI3K*, and* Akt* showed an expected upregulation after JHA application, whereas* 4E-BP* expression was decreased (Figure 7(b)), indicating that JHA application can increase the activity of the IIS pathway. Moreover, the fact that JHA application impaired the OXPHOS pathway also indicated an increased activity of the IIS pathway after JHA application because OXPHOS processes can be attenuated by abnormal nutritional signals (such as high insulin levels or a high-fat diet) in human skeletal muscle [54–57]. In combination with our observation of the JHA-induced modulation of carbohydrate metabolism, we speculate that JH might regulate carbohydrate metabolism to increase sugar levels in hemocytes, which could further activate the IIS pathway that is required for regulating glycolysis, lipid metabolism, and protein processing.

Insect larval molting is triggered by a pulse of 20E when the larvae grow to an appropriate body size under normal nutrient conditions [58, 59]. Thus, JHA-induced premature larval molting indicates that JHA application might accelerate the emergence of 20E pulse in the fourth larval instar. In fact, 20E biosynthesis was previously reported to be catalyzed by several cytochrome P450 enzymes [60]. Our data showed that the expression of two 20E-related genes,* Cyp314a1 *(*Shade*), which encodes a P450 enzyme for transforming inactive ecdysone into active 20E, and* EcR*, which encodes ecdysone receptor that is involved in 20E signaling, was significantly elevated following JHA application. This further confirmed that premature larval molting was likely resulted from the early emergence of 20E pulse after JHA application in the end of the fourth larval instar, which consequently contributed to shortening the developmental duration of the fourth larval instar and to subsequently reducing the body weight. In addition, we noted that compared to the early stages (12 and 24 Hat) after JHA application, a large number of genes showed more dramatic changes at the late stage of 48 Hat in expression levels, indicating that these JHA-modulated genes with dramatic expression changes may be just the indirect targets of JH, but the targets of 20E to a great extent. Furthermore, it has been known that insect metamorphosis with larval-pupal transition is also determined by a critical weight that is related to body size [61]. Given that the body weight of the JHA-induced superlarvae in the fifth larval instar was also reduced, we suggested that in the fifth larval instar, JHA-induced silkworm superlarvae did not reach to critical weight as same with that in the control, so it should experience an additional larval molting, which may be the same as the fourth instar of the control. Moreover, previous reports have demonstrated that 20E biosynthesis is also positively mediated by insulin signaling [32, 34, 62]. Together with our observation that genes involved in the IIS pathway were significantly upregulated after JHA application, we therefore suggest that JH may regulate 20E signaling by mediating basic metabolic activities and the nutrient-dependent IIS pathway. Further in vivo and in vitro experiments will be needed to test this hypothesis.

## 5. Conclusion

In this study, we profiled the genomewide alteration of gene expression in the integument of silkworm larvae after JHA application and found the cues about JH regulation on metabolic homeostasis, IIS pathway, and 20E signaling. Further studies should be conducted and may be helpful for deciphering the mechanism underlying the interaction between JH and 20E during silkworm larval growth.

## Supplementary Material

Supplementary Figure 1: Hierarchical clustering of a total of 8,670 genes expressed in the silkworm integument samples. A gene was considered to be expressed in a integument sample if its expression signal intensity was greater than 400 units.
Supplementary Table 1: All real time RT-PCR primers used in this study.
Supplementary Table 2: The list of 8,670 genes expressed in the silkworm integument samples. Integument-E: the integument sample treated with JHA methoprene. Integument-C: the integument sample treated with pure acetone.
Supplementary Table 3: The list of 2,143 JHA-modulated genes with 2.0-fold expression change after JHA application.
Supplementary Table 4: Correlation between microarray data and real time RT-PCR results. A good correlation was defined if the Pearson correlation coefficient (r) is greater than 0.7.
Supplementary Table 5: All KEGG pathways that JHA-modulated genes were involved in.
Supplementary Table 6: Coexpression classes of all JHA-modulated genes.
Supplementary Table 7: JHA-induced genes that contain the binding motifs of Kr-h1 in their upstream UTR regions.
Supplementary Table 8: JHA-modulated genes that are involved in each KEGG pathway.

## Abbreviations

JH: Juvenile hormone
JHA: Juvenile hormone analogue
20E: 20-Hydroxyecdysone
IIS: Insulin/insulin-like growth factor signaling
Hat: Hours after treatment
OXPHOS: Oxidative phosphorylation.
