# Supplementary material for: Microarray Analysis of the Juvenile Hormone Response in Larval Integument of the Silkworm, Bombyx mori
Source: Int J Genomics. 2014 Apr 6;2014:426025. doi: 10.1155/2014/426025 (PMC3997853; doi:10.1155/2014/426025)
Supplement: Supplementary file 1 — Supplementary Figure 1: Hierarchical clustering of a total of 8,670 genes expressed in the silkworm integument samples. A gene was considered to be expressed in a integument sample if its expression signal intensity was greater than 400 units. Supplementary Table 1: All real time RT-PCR primers used in this study. Supplementary Table 2: The list of 8,670 genes expressed in the silkworm integument samples. Integument-E: the integument sample treated with JHA methoprene. Integument-C: the integument sample treated with pure acetone. Supplementary Table 3: The list of 2,143 JHA-modulated genes with 2.0-fold expression change after JHA application. Supplementary Table 4: Correlation between microarray data and real time RT-PCR results. A good correlation was defined if the Pearson correlation coefficient (r) is greater than 0.7. Supplementary Table 5: All KEGG pathways that JHA-modulated genes were involved in. Supplementary Table 6: Coexpression classes of all JHA-modulated genes. Supplementary Table 7: JHA-induced genes that contain the binding motifs of Kr-h1 in their upstream UTR regions. Supplementary Table 8: JHA-modulated genes that are involved in each KEGG pathway. [file 426025.f1.zip › Supplementary materials/Fig.S1.pdf]

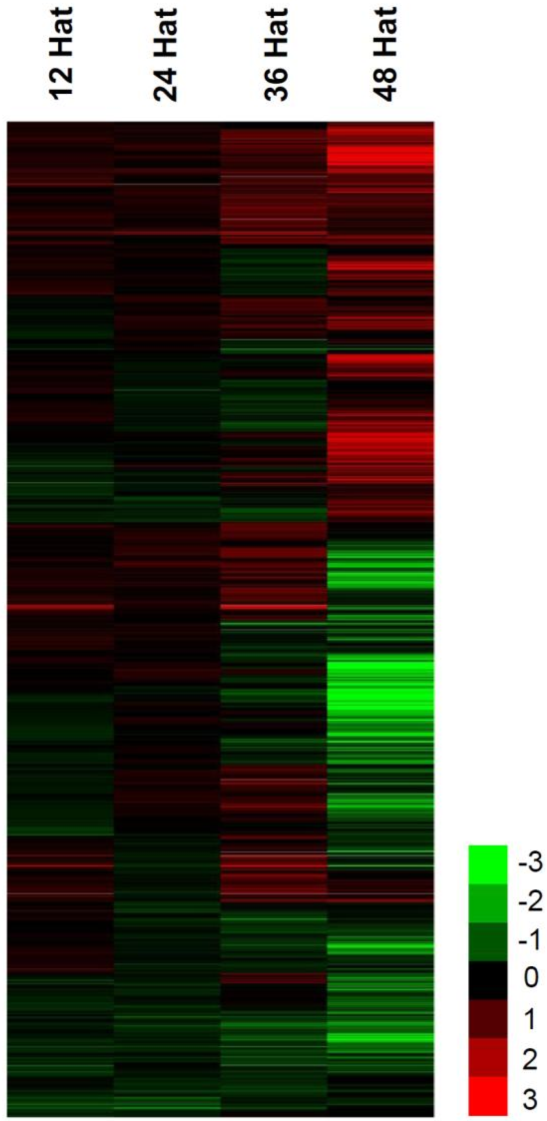

Fig. S1 Hierarchical clustering analysis of all expressed genes in the silkworm integument

Overall, 8,670 genes were expressed in the integuments of both JHA-induced silkworm superlarvae and controls. Hierarchical clustering of these genes is described in the Microarray analysis section of the Materials and Methods.
